# Valedictory

**Published:** 1882-06

**Authors:** Jno. Thad. Johnson


### Editorial.

### VALEDICTORY.

With the last issue of The Register my connection with it ceased. The earnest and careful editing of a journal demands more time and labor than I find convenient to devote to it.

The Register has my best wishes for that abundant success in the future which I know it will so well deserve under the management of Dr. Baird. I have had opportunity to appreciate his journalistic skill as well as his untiring energy.

It is pleasant to reflect that during the association just severed, the relations between the editors, and between them and the excellent publisher, Mr. Dickson, have been of a uniformly cordial character.

Jno. Thad. Johnson.

